# From learned helplessness to motor recovery: integrating intensive neurorehabilitation in poststroke spastic paresis—clinical insights from over 10 years of practice

**DOI:** 10.3389/fresc.2025.1644723

**Published:** 2026-01-30

**Authors:** Ibrahim Npochinto Moumeni

**Affiliations:** 1 Department of Physical Therapy & Physical Medicine, Faculty of Medicine and Pharmaceutical Sciences, University of Dschang, Dschang, West Region, Cameroon; 2 Department of Physical Medicine & Osteopathy, Regional Hospital of Bafoussam, Bafoussam, West Region, Cameroon; 3 Institute for Applied Neurosciences and Functional Rehabilitation (INAREF), Odza-Yaoundé, Cameroon; 4 Franco-African Center for Applied Rehabilitation and Health Sciences (CFARASS), Foumbot, West Region, Cameroon; 5 Department of Geriatrics and Gerontology, Sorbonne Université, Pitié-Salpêtrière Hospital, Paris, France; 6 Private Practitioner, Paris, France; 7 Neurorehabilitation, Faculty of Health Sciences, University of Parakou, Parakou, Benin; 8 French-Speaking African Society for Neurorehabilitation (SAFNeR), Parakou, Benin; 9 UREKIM – Research Unit in Physiotherapy and Physical Medicine, Faculty of Medicine and Pharmaceutical Sciences, University of Dschang, Dschang, West Region, Cameroon; 10 Centre de Recherche en Santé Humaine et Développement des Médicaments (CRESHDEM), Faculty of Medicine and Pharmaceutical Sciences, University of Dschang, Dschang, West Region, Cameroon

**Keywords:** stroke rehabilitation, spastic paresis, constraint-induced therapy, muscle plasticity, neuroplasticity, cross-cultural rehabilitation, therapeutic intensity, learned non-use

## Abstract

**Background:**

Poststroke spastic paresis represents a dual pathology combining neurological impairment and secondary muscle contracture, often perpetuated by learned non-use. Current rehabilitation approaches frequently address these components separately, limiting functional recovery potential.

**Methods:**

This perspective synthesizes over 10 years of clinical experience treating poststroke spastic paresis across European and sub-Saharan African settings, integrating constraint-induced movement therapy with progressive muscle-lengthening protocols. Clinical insights are drawn from over 300 patients treated in both resource-rich and resource-limited environments.

**Results:**

The integrated approach demonstrates superior outcomes compared with conventional therapy, with patients showing functional improvements even years after stroke. Key success factors include intensive training protocols, systematic antagonist muscle stretching, and patient-centered motivation strategies adapted to diverse cultural contexts. European validation and African implementation confirm universal applicability.

**Conclusion:**

Combining neuroplasticity-based interventions with muscle-targeted therapies offers a paradigm shift in poststroke rehabilitation. This approach proves effective across diverse healthcare settings, from high-technology European centers to resource-limited African hospitals, relying on intensive human intervention and evidence-based protocols.

## 1 Introduction

Methodological note: Patient testimonies presented throughout this article constitute anonymized composite illustrations constructed from recurrent clinical patterns observed in over 300 patients (poststroke) followed up between 2012 and 2024 across French and Cameroonian contexts. They aim to illustrate theoretical mechanisms without constituting primary research data.

“Doctor, look at my hand … it was like a closed fist, useless. My children told me to accept it. But you told me ‘we're going to wake up this hand’. Today, I can hold my grandchildren's hands again.” These words from Madame Sophie, a 67-year-old stroke survivor treated three years after stroke at the Regional Hospital of Bafoussam, encapsulate the transformative potential of intensive neurorehabilitation when hope seems lost.

Poststroke spastic paresis affects approximately 80% of stroke survivors, representing the leading cause of acquired motor disability in adults. However, the traditional understanding of this condition as solely neurological has evolved significantly over the past two decades. Current evidence reveals spastic paresis as a complex dual pathology: primary neurological impairment compounded by secondary muscle pathology induced by disuse and immobilization.

This perspective emerges from over 10 years of clinical practice spanning both European and sub-Saharan African settings, treating over 300 patients with chronic spastic paresis. The foundational training received during Master's studies in Movement Neurosciences and clinical internship in the UD (UNIVESRITY DIPLOMA) Neuro-rééducation du Mouvement at Henri Mondor University Hospital, France, under Prof. Jean-Michel Gracies' pioneering neurorehabilitation service, with guidance from Drs. Nicolas Bayle, Emilie Huntin, and Maud Pradines, provided the theoretical framework, while subsequent clinical practice in Cameroon demonstrated the universal applicability of these principles across diverse resource contexts.

The approach integrates constraint-induced movement therapy principles with systematic muscle-lengthening protocols, challenging the conventional separation of neurological and muscular interventions. This methodology, initially developed and validated in the high-technology environment of the Movement Analysis and Restoration Laboratory at Henri Mondor, has proven equally effective when adapted for resource-limited settings where advanced technologies are unavailable but human dedication is abundant.

This methodology addresses the learned helplessness phenomenon frequently observed in chronic stroke survivors, a form of acquired resignation exacerbated by systematic therapeutic pessimism documented in resource-limited health systems (1–3).

**Figure 1:**
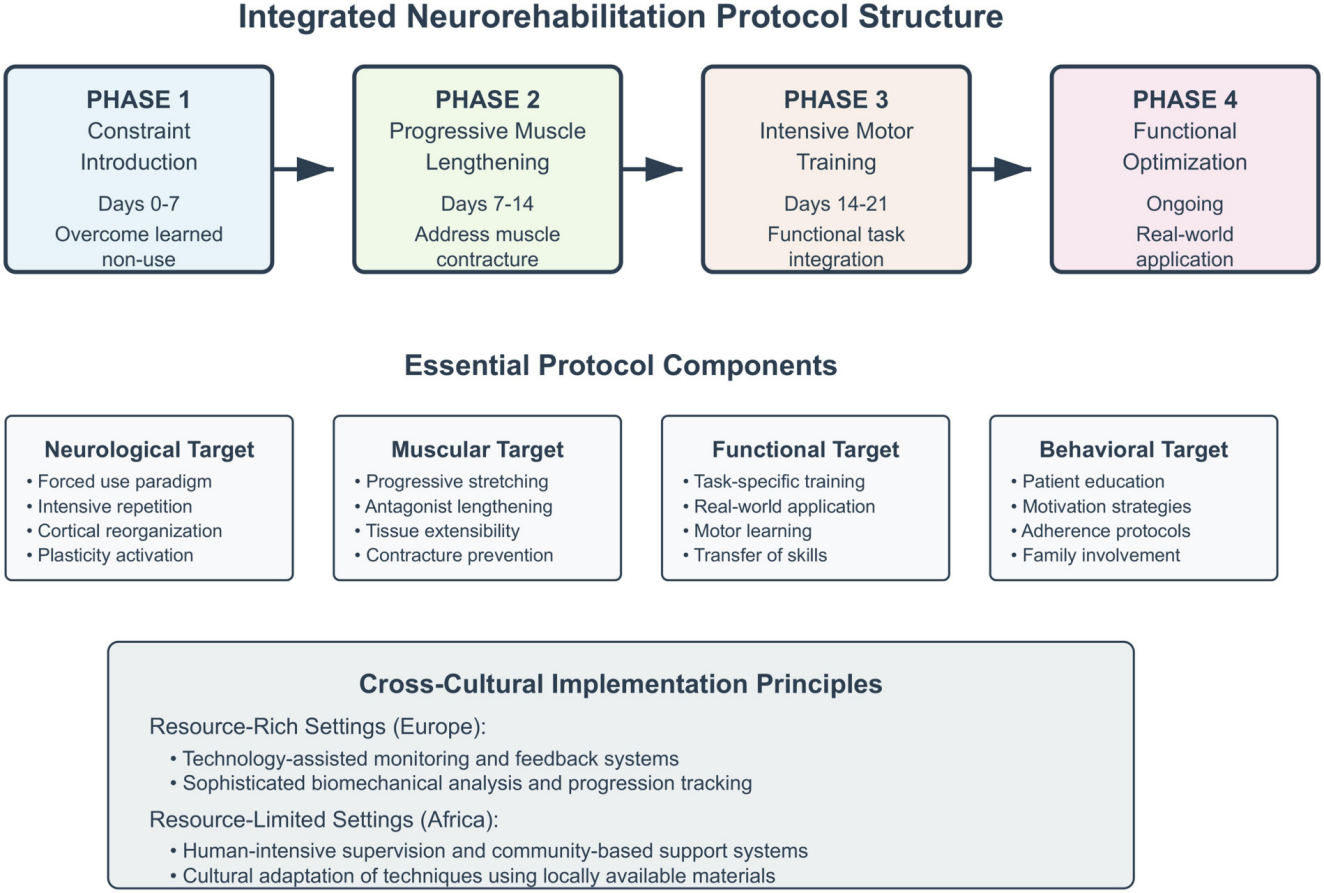
An integrated neurorehabilitation protocol structure showing the four-phase approach combining neurological, muscular, functional, and behavioral targets with adaptation strategies for diverse resource settings.

**Figure 2:**
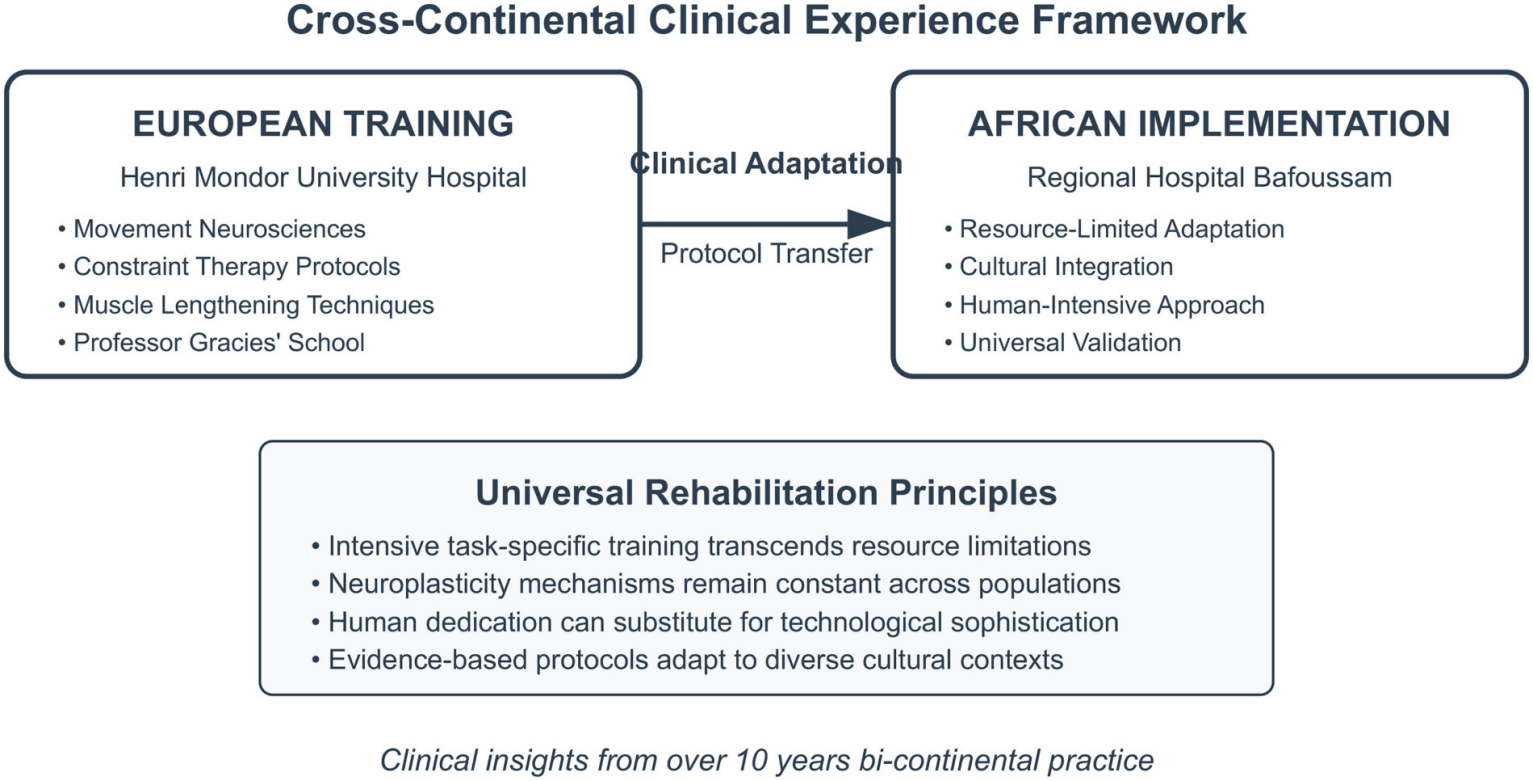
A cross-continental clinical experience framework showing the adaptation of intensive neurorehabilitation principles from European training to African implementation, demonstrating universal applicability across diverse resource contexts.

The theoretical foundation rests on three pillars derived from the Gracies school of thought: understanding learned non-use as a reversible phenomenon through forced-use paradigms, recognizing muscle contracture as the primary limiting factor in spastic paresis recovery, and leveraging neuroplasticity through intensive, task-specific training protocols (4, 5) (Figure 1). This integrated framework offers new possibilities for stroke survivors globally, particularly in regions where rehabilitation resources are scarce but human commitment can substitute for technological limitations (see Figure 2).

## 2 The dual pathology of spastic paresis: from theory to practice

### 2.1 Theoretical framework: the Gracies model

The conceptual understanding of spastic paresis as a dual pathology stems from pioneering work at the Movement Analysis and Restoration Laboratory, where Prof. Jean-Michel Gracies and his team, Drs. Nicolas Bayle, Emilie Huntin, and Maud Pradines, have systematically documented the complex interplay between neurological dysfunction and secondary muscle changes. This model fundamentally challenges traditional rehabilitation approaches that focus primarily on neurological recovery while neglecting the profound muscular adaptations occurring within hours of stroke onset (1, 2, 6).

**Figure 3:**
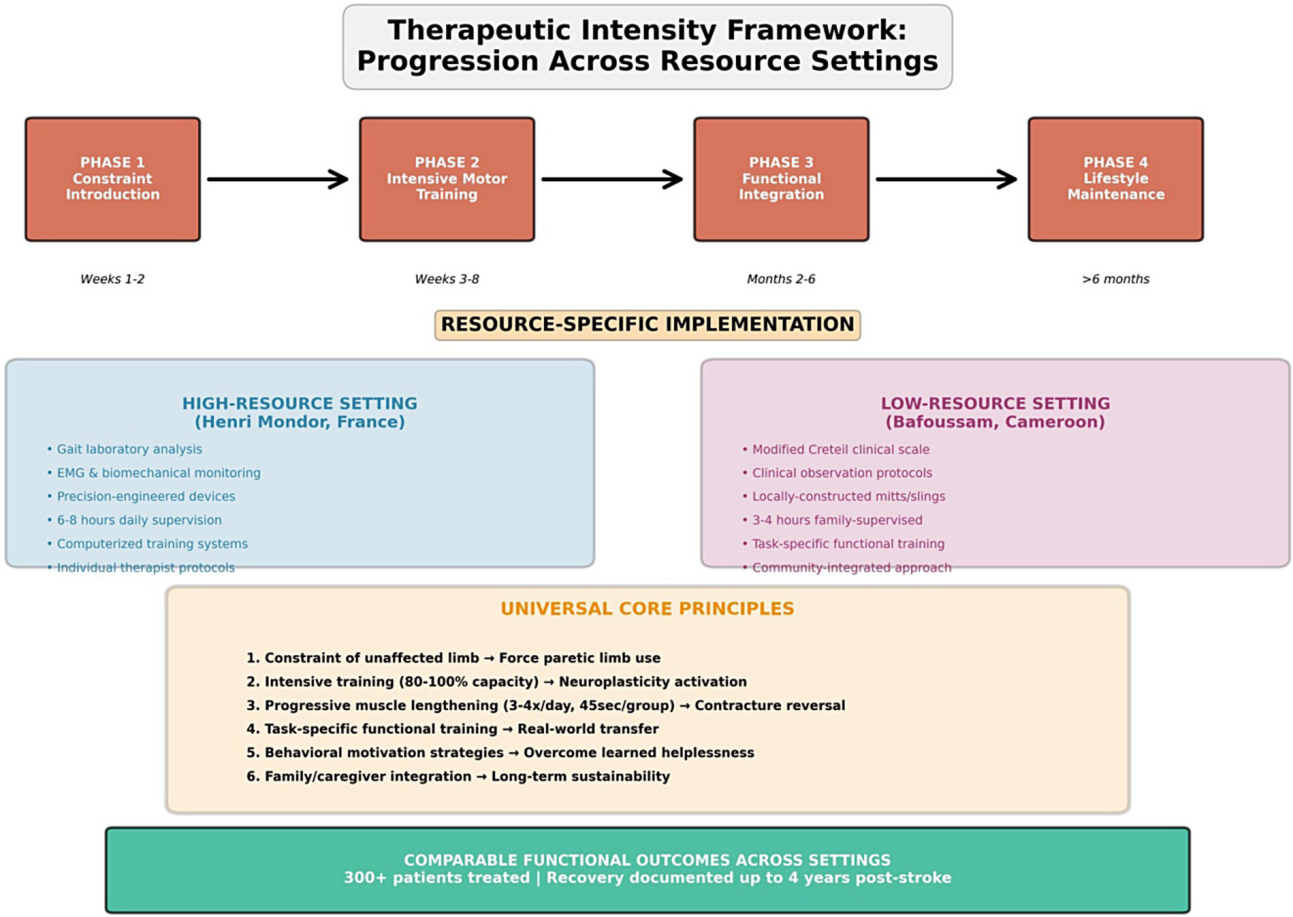
A therapeutic intensity framework: progression across resource settings. The figure illustrates the universal four-phase therapeutic progression (Phase 1: Constraint Introduction, Phase 2: Intensive Motor Training, Phase 3: Functional Integration, Phase 4: Lifestyle Maintenance) with resource-specific implementation strategies. Blue panel: a high-resource European setting (Henri Mondor) utilizing advanced technology. Purple panel: a low-resource African setting (Bafoussam) utilizing family-based approaches. Orange panel: universal core principles applicable across all resource contexts, emphasizing that comparable functional outcomes are achievable through human-intensive intervention regardless of technological availability.

Clinical observation across both European and African settings reveals that patients begin developing what Gracies termed “spastic myopathy” as early as 48–72 h after stroke, characterized by rapid loss of muscle extensibility, sarcomere reduction, and increased non-extensible collagen deposition. This phenomenon occurs regardless of the healthcare setting, confirming its fundamental biological nature rather than care-related factors (6, 7, see Figure 3).

This dual neurological and muscular pathology (8, 9) constitutes the rational foundation of our integrated approach. Recent studies confirm that the intensity of therapeutic intervention directly modulates functional recovery, regardless of postlesional delay (10). Our transcontinental experience, documented over more than a decade (2, 5), demonstrates that neuroplastic mechanisms can be activated even in the absence of sophisticated technologies.

Monsieur Paul, a 54-year-old mechanic treated in Bafoussam who eventually returned to work 18 months after his stroke, initially presented with severe finger flexor contractures. His wife recalled: *“*His hand was completely closed, the fingers curled so tight we couldn't clean his palm properly. The doctors said it was brain damage, nothing could be done.” However, systematic muscle lengthening combined with intensive finger training, based on protocols learned at Henri Mondor, gradually restored function that many deemed permanently lost.

### 2.2 The vicious cycle of disuse: European insights and African validation

The pathophysiology of spastic paresis involves a self-perpetuating cycle where initial paresis leads to disuse, which triggers muscle contracture, subsequently increasing apparent spasticity and further limiting movement. This cycle, extensively documented in European populations, proves equally evident in African patients, confirming its universal nature across genetic and cultural backgrounds.

During clinical training and Master's internship at Henri Mondor, the sophisticated movement analysis laboratory allowed precise documentation of this cycle through quantitative gait analysis and biomechanical assessments. In African settings, which lacked such technology, clinical observation and the modified Créteil scale provided equally valuable insights into the same pathophysiological processes.

This cycle is particularly evident in upper limb presentation, where patients quickly learn to compensate with the unaffected side, essentially “forgetting” to use the paretic limb—a phenomenon that Taub and colleagues first described as “learned non-use.” Clinical experience across both continents demonstrates that breaking this cycle requires simultaneous intervention on multiple fronts, regardless of available technology.

### 2.3 Neuroplasticity windows: universal principles

Contrary to traditional beliefs about limited recovery windows, clinical evidence from both European and African practices consistently demonstrates neuroplasticity potential years after stroke onset. Madame Christine, treated in Cameroon but following protocols validated in France, initially resisted constraint therapy. She later reflected: “That glove that prevented me from using my good hand—it was my strictest trainer. I hated it at first, but it forced my weak hand to remember how to work.”

Recent advances in neuroscience confirm that cerebral plasticity persists well beyond critical developmental periods (11, 12), offering therapeutic opportunities even in chronic poststroke phases (1, 10). The key insight from extensive bicontinental clinical practice is that accessing neuroplastic mechanisms requires intensity levels rarely achieved in conventional rehabilitation. This principle, first observed during Master's training and clinical internship with Prof. Gracies' team, has proven consistent across diverse patient populations and healthcare environments.

## 3 Integrated therapeutic approach: cross-cultural implementation

### 3.1 Constraint-induced movement therapy: from Henri Mondor to Bafoussam

The constraint-induced movement therapy protocols learned at Henri Mondor required significant adaptation for implementation in resource-limited African settings. Standard protocols requiring 6–8 h daily supervision with sophisticated monitoring equipment proved impractical, necessitating innovative adaptations, while maintaining core therapeutic principles.

The adapted approach maintains essential elements inspired by seminal constraint-induced movement therapy research (13–15): constraint of the unaffected limb, intensive training of the paretic limb, and behavioral techniques to overcome learned non-use. These core principles, adapted to local contexts (2, 7), ensure therapeutic fidelity, while maximizing cultural acceptability. However, implementation strategies differ significantly. In European settings, constraint devices are precision-engineered with force sensors and compliance monitoring. In African settings, simple mitts or slings constructed locally prove equally effective when properly applied.

The psychological component, emphasized throughout training with Drs. Bayle, Huntin, and Pradines, proves as crucial as physical constraint regardless of setting. Patients require careful preparation and motivation strategies adapted to cultural contexts. In sub-Saharan African settings, extended family involvement becomes essential, with relatives trained to provide encouragement and monitor home exercises. Traditional healing concepts of gradual restoration often align well with constraint therapy principles, facilitating patient acceptance.

### 3.2 Progressive muscle lengthening: universal biomechanics

Systematic muscle stretching protocols, refined through collaboration with the Henri Mondor team, constitute the foundation of the integrated approach. The biomechanical principles remain constant across settings: targeting specifically the antagonist muscles that limit functional movement through prolonged stretching (minimum 45 s per muscle group) performed 3–4 times daily, with intensity sufficient to reach mild discomfort thresholds.

Monsieur Jean, a 62-year-old farmer from the West Region of Cameroon, described his experience: “My muscles were like ropes tied too tight. These stretches hurt at first, but now I can hold my hoe again.” His case illustrates the universal importance of patient education regarding stretch discomfort vs. harmful pain, principles emphasized throughout European training but equally applicable in African agricultural communities.

The muscle-lengthening protocols require months of consistent application, with measurable improvements typically emerging after 8–12 weeks regardless of setting. This timeline, validated through extensive European research, proves consistent in African populations despite genetic, nutritional, and environmental differences.

### 3.3 Intensive motor training: task-specific adaptation

Motor training follows task-specific principles developed at Henri Mondor but adapted to local functional requirements. European protocols often emphasize computer use, fine writing tasks, and functional movement in an industrial setting. African adaptations incorporate functional movement in a farm setting, traditional craft activities, and domestic tasks specific to local contexts.

In agricultural communities, exercises incorporate hoe handling, seed planting movements, and harvest activities. For urban African patients, training emphasizes writing, food preparation, or commercial activities. This functional relevance enhances motivation and facilitates transfer to real-world activities, confirming principles learned during European training.

The intensity principle, fundamental to the Gracies approach, cannot be overstated regardless of setting. Effective motor learning requires pushing patients beyond their comfort zones, demanding movements at 80%–100% of current capacity. This intensity-dependent neuroplasticity activation, supported by recent evidence demonstrating superior outcomes with high-intensity protocols even in acute phases (10), relies on progressive activation of cortical and muscular plasticity mechanisms (4, 5). This approach challenges both patients and therapists, requiring careful balance between therapeutic aggression and safety considerations across all cultural contexts.

## 4 Clinical implementation framework: bridging continental practices

### 4.1 Assessment protocols: from laboratory to bedside

Initial evaluation employs the modified Créteil scale (five-stage evaluation) developed through collaborative work between Henri Mondor University Hospital and University of Créteil Paris 13. This assessment framework quantifies spasticity, contracture, and weakness components separately, providing objective measures for treatment planning and outcome monitoring regardless of available technology.

In European settings, this assessment complements sophisticated biomechanical analysis including gait laboratories, electromyographic studies, and computerized movement analysis. In African settings, the clinical scale provides equivalent prognostic and monitoring capabilities when properly applied, demonstrating the robustness of the clinical assessment methods developed by Prof. Gracies' team.

Patient selection criteria remain consistent across settings: presence of minimal voluntary movement (even trace muscle activation), absence of severe cognitive impairment preventing instruction following, and most importantly, patient motivation and family support. Motivation assessment, emphasized throughout training at Henri Mondor, often proves more predictive of success than initial motor capacity regardless of the healthcare environment.

### 4.2 Progression protocols: universal staging

Treatment progression follows structured frameworks developed at Henri Mondor but adapted for local implementation capabilities. The fundamental staging remains constant: constraint introduction and basic stretching protocols (weeks 1–2), intensive motor training with progressive difficulty increases (weeks 3–8), and functional task integration with independence development (months 2–6).

Critical success factors identified through both European and African practices include regular reassessment and protocol adjustment based on individual response patterns. Some patients respond rapidly to constraint therapy, while others require extended muscle-lengthening phases before motor training becomes effective. This flexibility in approach timing, while maintaining intensity principles, optimizes individual outcomes across diverse populations.

The bicontinental experience reveals that therapeutic principles transcend cultural and resource boundaries when properly adapted. Success depends more on understanding fundamental pathophysiology and maintaining therapeutic intensity than on sophisticated equipment or specific cultural approaches.

### 4.3 Home exercise integration: cultural adaptation

Sustainability requires effective home exercise programs that patients can maintain independently regardless of socioeconomic status. Families receive training in stretching techniques, constraint application, and motivation strategies adapted to local contexts. Simple exercise equipment constructed from local materials (resistance bands, weighted objects, modified tools) ensures program accessibility.

European patients often prefer individual exercise programs with technological monitoring. African patients typically benefit from family-integrated approaches with community support systems. However, both populations require detailed exercise logs that provide psychological reinforcement through visible progress documentation, confirming universal psychological principles underlying motor learning.

## 5 Outcomes and clinical insights: cross-continental validation

### 5.1 Functional recovery patterns: universal biology

Over 10 years of clinical experience across European and African populations reveals consistent recovery patterns among patients completing integrated protocols. Upper limb improvements typically emerge in proximal-to-distal progression, with shoulder and elbow function recovering before hand dexterity. Lower limb recovery often shows more rapid progression, reflecting the bilateral cortical representation of walking functions.

These patterns, first observed during training at Henri Mondor with patients of diverse European backgrounds, prove remarkably consistent in African populations despite genetic, nutritional, and environmental differences. This consistency confirms the fundamental biological nature of stroke recovery rather than culturally specific phenomena.

Most encouraging are cases of late recovery in patients initially deemed hopeless across both continents. Madame Françoise, treated in Cameroon 4 years after stroke using protocols learned in France, regained sufficient hand function to resume her tailoring work. Her case exemplifies the potential for meaningful recovery when intensive protocols are properly applied, regardless of setting or initial prognosis.

### 5.2 Resource adaptation: innovation through limitation

Resource limitations in African settings present both challenges and opportunities compared with well-equipped European centers. While advanced technology remains unavailable, human resources often exceed those in developed countries. Extended family support systems provide natural constraint enforcement and exercise supervision, often lacking in individualistic European societies.

The experience gained at Henri Mondor, with access to sophisticated rehabilitation robotics and computerized training systems, initially suggested that such technology was essential for optimal outcomes. However, African practice demonstrated that human-intensive interventions could achieve comparable outcomes when properly structured and consistently applied, challenging assumptions about technology dependence in rehabilitation.

Cultural factors significantly influence outcomes but in ways that transcend simple geographic boundaries. Traditional beliefs about disability and recovery vary widely within both European and African populations, requiring careful individual adaptation of therapeutic messages regardless of continental location.

### 5.3 Long-term sustainability: universal challenges

Follow-up data spanning 3–5 years’ post-treatment across both European and African populations reveal that functional gains sustain when patients continue modified home programs. However, complete cessation of stretching protocols typically results in gradual contracture recurrence, emphasizing the chronic nature of muscle management requirements regardless of the initial treatment setting.

The most successful long-term outcomes occur when patients integrate therapeutic exercises into daily routines rather than viewing them as temporary medical interventions. This paradigm shift from treatment to lifestyle modification proves crucial for sustained maintenance of benefits across all cultural contexts, confirming insights gained during European training and validated through African practice.

## 6 Implications for global rehabilitation: lessons from two continents

### 6.1 Addressing resource disparities: technology vs. intensity

The bicontinental experience offers unique insights into addressing global rehabilitation disparities. High-technology European approaches achieve excellent outcomes but remain economically inaccessible for most global stroke survivors. Human-intensive interventions can achieve comparable outcomes when properly structured and consistently applied, offering hope for global stroke care equity.

Training local healthcare workers in intensive rehabilitation principles creates sustainable capacity building that transcends technology transfer limitations. Unlike equipment-dependent approaches, skill-based knowledge remains permanently within communities, providing ongoing benefit multiplication effects that extend far beyond individual patient encounters.

The collaboration between Henri Mondor's sophisticated research environment and Bafoussam's resource-limited clinical setting demonstrates that fundamental rehabilitation principles transcend resource availability when human dedication substitutes for technological limitations.

### 6.2 Challenging recovery assumptions: universal plasticity

Clinical experience across diverse populations consistently challenges assumptions about recovery limitations and optimal intervention timing. Patients showing meaningful functional improvements years after stroke, regardless of continental location or treatment setting, demonstrate that neural plasticity windows remain accessible when appropriate levels of therapeutic intensity are maintained (16–18).

The economic implications are substantial across all healthcare systems. Extended rehabilitation potential justifies continued investment in chronic stroke care, contradicting cost-effectiveness models based on limited recovery assumptions (19, 20). When properly implemented, intensive rehabilitation generates long-term independence gains that offset initial investment costs in both European and African healthcare economies (12, 15, 19).

### 6.3 Research and development priorities: global collaboration

Four priority research axes emerge from this transcontinental experience: First, dose–response optimization studies are needed to determine optimal intensity thresholds (repetitions per day and session duration) according to lesional profile and poststroke delay (10). Our clinical protocols, including structured 45-s cycle interventions (6), require multicenter validation across diverse populations.

Second, cultural adaptation of constraint-induced movement therapy demands systematic investigation. Family adherence models to therapeutic constraints must be compared between collectivist African and individualist European contexts, with the development of validated psychometric tools for cross-cultural implementation (7).

Third, long-term outcome registries in resource-limited settings would document the sustainability of functional gains beyond 5 years in the absence of sophisticated monitoring technologies (2, 3). Such registries could identify factors predicting maintenance of recovery in diverse healthcare environments.

Fourth, accessible plasticity biomarkers merit investigation. Identification of simple clinical markers—joint amplitude, gesture velocity, functional scores—predictive of neuroplastic reactivation would facilitate large-scale deployment of intensive protocols (1, 4, 5). Such markers could guide individualized intensity calibration across resource contexts.

International collaboration models emerging from Henri Mondor–Bafoussam partnerships provide templates for global replication, comparing outcomes across diverse cultural and resource contexts while identifying fundamental therapeutic principles that transcend geographic boundaries.

## 7 Conclusion

The integration of constraint-induced movement therapy with progressive muscle lengthening represents a paradigm shift in poststroke rehabilitation, addressing both neurological and muscular components of spastic paresis through intensive, human-centered interventions. Over 10 years of clinical experience across European and African settings demonstrates that meaningful functional recovery remains possible years after stroke onset when appropriate levels of therapeutic intensity are maintained, regardless of available technology.

This approach offers particular promise for global stroke care equity, proving that fundamental rehabilitation principles transcend resource limitations when human dedication substitutes for technological sophistication. The theoretical foundation developed at Henri Mondor University Hospital under Prof. Gracies' leadership provides universal applicability when adapted to local contexts and resource availability.

In settings where advanced technology is lacking, it is rigor, clinical awareness, and therapeutic creativity that become the true instruments of care. This human-intensive approach invites us to move beyond dependence on sophisticated equipment to reconnect with grounded, evidence-based rehabilitation that remains accessible across diverse healthcare environments. The essence of effective neurorehabilitation lies not in the sophistication of our tools, but in the precision of our understanding and the intensity of our therapeutic commitment.

Success depends on understanding spastic paresis as a dual pathology requiring simultaneous neurological and muscular intervention, maintaining therapeutic intensity beyond conventional comfort levels across all cultural contexts, and adapting implementation protocols to local resource and cultural constraints, while preserving essential therapeutic principles. The testimonies of hundreds of patients across two continents who have regained functional independence challenge traditional assumptions about stroke recovery limitations.

From the sophisticated laboratories of Henri Mondor to the resource-limited wards of Bafoussam Regional Hospital, we have learned that the window for meaningful stroke recovery never truly closes. It simply requires the right approach—intensive, integrated intervention delivered with unwavering commitment to human potential for recovery and adaptation. When technology is scarce, human expertise, clinical rigor, and therapeutic dedication become our most powerful rehabilitation tools.

Madame Sophie's journey from despair to renewed purpose, Monsieur Paul's return to meaningful work, and countless other recovery stories exemplify the transformative potential of intensive rehabilitation when hope, science, and human determination converge. These experiences remind us that effective neurorehabilitation transcends geographic boundaries and resource limitations when fundamental principles are properly understood and human-centered approaches replace technology-dependent interventions.

As incidents of global stroke continue to rise, particularly in developing countries with limited rehabilitation infrastructure, the collaboration between European research excellence and African clinical innovation demonstrates that effective rehabilitation can reach all stroke survivors. This partnership model validates that meaningful recovery is not a privilege of wealthy healthcare systems, but a universal right achievable through dedicated clinical practice and evidence-based human intervention.

The evidence is clear across continents and cultures: neuroplasticity knows no borders, and the capacity for human motor recovery transcends economic circumstances. In contexts where resources are limited, it is not the poverty of means that hinders innovation, but forgetting the clinical fundamentals: listening, adapting, repeating, and believing in the intelligence of precisely applied therapeutic intervention.

### 7.1 A message to future practitioners

Do not wait for a fully equipped rehabilitation center to begin healing. Learn to read movement dysfunction with your eyes, not sensors. Learn to assess muscle tone with your hands, not algorithms. You are the inheritors of a global rehabilitation tradition that should not seek merely to imitate, but to inspire. Human-centered rehabilitation is not disguised poverty, but thoughtfully applied richness of clinical understanding.

This work stands as a testament to the fact that when sophisticated equipment is unavailable, the sophisticated clinician becomes the instrument of recovery—combining scientific rigor with therapeutic creativity, European training with African innovation, and technological understanding with fundamentally human care.

## Data Availability

The original contributions presented in the study are included in the article/Supplementary Material, further inquiries can be directed to the corresponding author/s.
