# Supplementary material for: From learned helplessness to motor recovery: integrating intensive neurorehabilitation in poststroke spastic paresis—clinical insights from over 10 years of practice
Source: Front Rehabil Sci. 2026 Jan 30;6:1644723. doi: 10.3389/fresc.2025.1644723 (PMC12901396; doi:10.3389/fresc.2025.1644723)
Supplement: Supplementary file 1 [file Table1.docx]

**SUPPLEMENTARY FILES RECOMMANDÉS :**

**Supplementary File 1: Comparative Framework**

**"Supplementary Table S1: Evolution of Post-Stroke Rehabilitation Paradigms"**

- Tableau comparatif détaillé :
  - Époque → Approche → Mécanismes → Outcomes
  - 1950s-1970s : Passive mobilization
  - 1980s-1990s : Active conventional therapy
  - 2000s-2010s : Task-specific training
  - 2010s-Present : Intensive constraint therapy

**Supplementary File 2: Clinical Protocols**

**"Supplementary Material S1: Detailed Implementation Protocols"**

- Protocoles step-by-step pour :
  - Constraint-induced movement therapy
  - Progressive muscle lengthening
  - Assessment scales (Créteil, Fugl-Meyer)
  - Adaptation pour contextes resource-limited

**Supplementary File 3: Evidence Summary**

**"Supplementary Table S2: Systematic Evidence Review"**

- Méta-analyse des études clés :
  - Auteurs, année, population, intervention, outcomes
  - Niveaux de preuve (Ia, Ib, IIa, etc.)
  - Effect sizes et confidence intervals

**Supplementary File 4: Cross-Cultural Adaptation**

**"Supplementary Material S2: Resource-Setting Adaptation Guidelines"**

- Framework d'adaptation :
  - High-resource vs low-resource settings
  - Equipment alternatives
  - Staff training requirements
  - Cost-effectiveness analysis

**Supplementary File 5: Future Directions**

**"Supplementary Figure S1: Translational Research Pipeline"**

- Flowchart recherche → clinique :
  - Basic neuroscience discoveries
  - Preclinical validation
  - Clinical trials
  - Implementation strategies
